# Testing entropy-based search strategies for a visual classification task

**DOI:** 10.1186/1471-2202-13-S1-P109

**Published:** 2012-07-16

**Authors:** Liliya Avdiyenko, Nils Bertschingen, Juergen Jost

**Affiliations:** 1 Max Planck Institute for Mathematics in the Sciences, Leipzig, 04103, Germany

## 

There is experimental evidence that saccades during visual search preferentially target locations that contain task-relevant information [1]. However, the question remains what kind of strategy people use to decide what is relevant for a task. Do we use simple heuristics or complex algorithms based on the ideas of information theory? For example, for a shape-learning and -matching task sequential entropy-minimization was successfully used to predict human fixations [2]. Inspired by this fact, we test three entropy-based strategies for sequential location (image patches) selection during a visual classification task.

**Figure 1:**
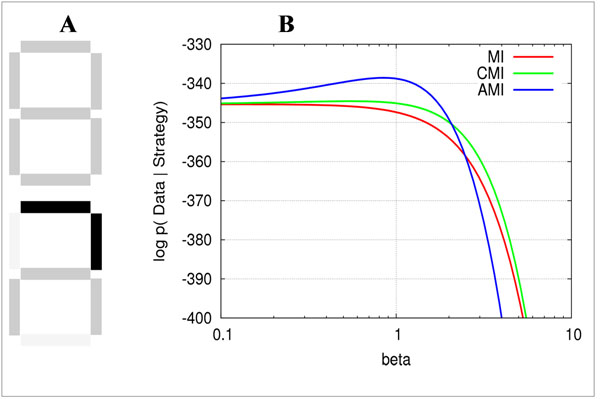
**A.** Examples of covered and partially uncovered digits. **B.** Log-likelihoods of the observed data given different strategies for a single subject.

In our experiments, we used a more constrained setup than eye-tracking, were the subject had to click on an image location explicitly. During the experiment, images of clock digits consisting of seven patches at different locations are presented. Initially all patches are covered and shown as a gray outline (Figure 1A). Patches can be uncovered by clicking at them. The task is to uncover a minimal number of patches that, in the subject's opinion, help to identify the digit. The presentation stops when the digit is unambiguously identified (Figure 1 A bottom).

Determining the task-relevant parts of a visual scene is closely related to the problem of feature selection in machine learning. Correspondingly, we tested to what extend human behavior can be explained in terms of feature selection criteria.

The first strategy (MI, Mutual Information), is considered as a heuristic. It simply ranks all locations according to the mutual information they provide about digit identity . The second strategy (CMI, Conditional Mutual Information) takes into account high-order dependencies between locations, i.e. selects those that are both informative and non-redundant with respect to the already selected locations. The third strategy (AMI, Adaptive conditional Mutual Information) is an adaptive version of CMI, which takes into account also observed values of the already attended locations, suggesting that every next decision depends on what one has seen on the previous steps. Thus, in contrast to MI and CMI, the optimal location sequence is different for each image.

We compare these strategies with respect to their explanatory power of the observed behavioral data . For this, we turn them into generative models of patch sequences. Each model assumes that the next patch is chosen as softly maximizing the strategy-specific information about the image class. The softmax function is parametrized with β. For low values of β all sequences are equally likely, i.e. the subject acts randomly, whereas a high β concentrates the probability mass on sequences which select the most informative patches – according to each strategy – on each step. Preliminary results show that the behavior of most subjects is best explained by the AMI strategy.

Our clicking experiment provide evidence that for a visual classification task people are able to employ quite complex entropy-based search strategies. We found in particular that, even though it is more computationally demanding, most people act adaptively, i.e. take into account image-specific information.
